# SARS-CoV-2-Associated ssRNAs Activate Human Neutrophils in a TLR8-Dependent Fashion

**DOI:** 10.3390/cells11233785

**Published:** 2022-11-26

**Authors:** Elisa Gardiman, Francisco Bianchetto-Aguilera, Sara Gasperini, Laura Tiberio, Matteo Scandola, Virginia Lotti, Davide Gibellini, Valentina Salvi, Daniela Bosisio, Marco A. Cassatella, Nicola Tamassia

**Affiliations:** 1 General Pathology Section, Department of Medicine, University of Verona, 37134 Verona, Italy; 2 Department of Molecular and Translational Medicine, University of Brescia, 25123 Brescia, Italy; 3 Microbiology Section, Department of Diagnostic and Public Health, University of Verona, 37134 Verona, Italy

**Keywords:** neutrophils, TLR8, SARS-CoV-2, ssRNA, RNA-seq, neutrophil extracellular trap, COVID-19

## Abstract

COVID-19 disease is characterized by a dysregulation of the innate arm of the immune system. However, the mechanisms whereby innate immune cells, including neutrophils, become activated in patients are not completely understood. Recently, we showed that GU-rich RNA sequences from the SARS-CoV-2 genome (i.e., SCV2-RNA1 and SCV2-RNA2) activate dendritic cells. To clarify whether human neutrophils may also represent targets of SCV2-RNAs, neutrophils were treated with either SCV2-RNAs or, as a control, R848 (a TLR7/8 ligand), and were then analyzed for several functional assays and also subjected to RNA-seq experiments. Results highlight a remarkable response of neutrophils to SCV2-RNAs in terms of TNFα, IL-1ra, CXCL8 production, apoptosis delay, modulation of CD11b and CD62L expression, and release of neutrophil extracellular traps. By RNA-seq experiments, we observed that SCV2-RNA2 promotes a transcriptional reprogramming of neutrophils, characterized by the induction of thousands of proinflammatory genes, similar to that promoted by R848. Furthermore, by using CU-CPT9a, a TLR8-specific inhibitor, we found that SCV2-RNA2 stimulates neutrophils exclusively via TLR8-dependent pathways. In sum, our study proves that single-strand RNAs from the SARS-CoV-2 genome potently activate human neutrophils via TLR8, thus uncovering a potential mechanism whereby neutrophils may contribute to the pathogenesis of severe COVID-19 disease.

## 1. Introduction

From the end of 2019, the world has suffered from the COVID-19 pandemic, which to date has caused more than six million deaths and has involved over 600 million people worldwide [1]. The COVID-19 causative agent is a virus belonging to the Coronaviridae family and is named SARS-CoV-2 [2,3]. It contains a positive single-strand RNA (ssRNA+) genome of ~30 kb [4], which shares a high similarity to those of SARS-CoV and MERS-CoV, which both caused outbreaks of respiratory syndromes in 2002 and 2013, respectively [2]. COVID-19 includes heterogeneous diseases, developing from mainly asymptomatic and/or mild to severe courses, with life-threatening conditions in 10–20% of symptomatic patients [5]. The most common symptoms of COVID-19 consist of flu-like features, such as fever, fatigue, and dry cough [6,7]. The elderly (>60 years), or people with comorbidities, are more likely to develop severe disease requiring intensive care, characterized by acute lung injury, acute respiratory distress syndrome (ARDS), sepsis-like manifestations, and multiorgan failure [8,9]. Although the precise reasons why some COVID-19 patients develop severe disease have not been clarified yet, the systemic inflammation provoked by an excessive and uncontrolled production of pro-inflammatory cytokines (the so called “cytokine storm”) certainly represents a major mechanism [10]. Even though the impact of SARS-CoV-2 burden is currently reduced thanks to vaccine development and administration, COVID-19 still represents a serious threat to global health.

Neutrophils are the first leukocytes that migrate from blood to infected sites, retained mainly to fight bacterial infections. However, recent studies indicate that human neutrophils may also react to viral infections [11], as they not only express numerous PRRs known to recognize PAMPs of viral origin, including TLR8, MDA5, RIGI, and IFI16 [12,13,14], but also because they promptly respond to type I interferons [15,16,17]. Moreover, we uncovered that small molecules mimicking the action of ssRNA, acting via TLR8 (i.e., R848, CL075 or VTX-2337), dramatically reprogram human neutrophils at the transcriptomic level through mechanisms involving chromatin remodeling and activation of transcription factors such as NF-kB, AP-1, and OCT2 [18,19]. As mentioned, natural ligands of TLR8 typically consist of GU-rich ssRNA [20], which is noticeable since we recently demonstrated that SARS-CoV-2 genome contains several GU-rich sequences [21]. Two of these sequences, namely SCV2-RNA1 and SCV2-RNA2, were shown to be highly immunostimulatory for human plasmacytoid dendritic cells (pDCs) and classical DCs, leading to their production of, respectively, high levels of type I interferons and pro-inflammatory cytokines [21]. Moreover, we also reported that in mice these GU-rich sequences contribute to the activation and maturation in vivo of splenic pDCs, cDC1, and cDC2 [21]. In addition, ssRNAs induced MyD88-dependent expression of pro-inflammatory cytokines (TNF-α, IL-1β, and IL-6), IFN-α and cytotoxic mediators in the lung, in turn, leading to immune cell infiltration [21]. 

In this study, we investigated whether human neutrophils respond to these SARS-CoV-2 GU-rich sequences [21]. We thus explored whether SCV2-RNAs affect cytokine release, degranulation, phenotype, neutrophil extracellular trap (NET) formation, and transcriptome of human neutrophils.

## 2. Materials and Methods

### 2.1. Cell Isolation and Stimulation

Neutrophils were isolated from buffy coats of healthy donors using Ficoll-Paque ^TM^ PLUS (Cytiva, Uppsala, Sweden) gradient centrifugation (30 min at RT, 400× *g*) and manipulated under endotoxin-free conditions. The granulocytic fraction was subjected to dextran sedimentation followed by erythrocyte hypotonic lysis. Further purification of neutrophils (approximately 99.7% purity) was obtained using the EasySep neutrophil enrichment kit (StemCell Technologies, Vancouver, BC, Canada) [22]. Neutrophils were then suspended at 5 × 10^6^/mL in RPMI 1640 medium (Thermo Fisher Scientific, Waltham, MA, USA) containing 10% FBS (with <0.5 EU/mL endotoxin, Sigma-Aldrich, Saint Louis, MO, USA). For stimulation of cells with RNA oligonucleotides we complexed RNA with DOTAP Liposomal Transfection Reagent (Roche, Basel, Switzerland) as previously described [21]. Briefly, 2.5–10 μg RNA resuspended in 50 μL HBS buffer (20 mM HEPES, 150 mM NaCl, pH 7.4) was combined with 100 μL DOTAP solution (30 μL DOTAP plus 70 μL HBS buffer) and incubated for 15 min at RT. The following RNA oligonucleotides, used also in our previous study [21], were synthesized by Integrated DNA Technologies (IDT, Coralville, IA, USA): SCV2-RNA1 5′-UGCUGUUGUGUGUU*U-3′; SCV2-RNA2 5′-GUGUGUGUGUUCUGUUAUU*G-3′; SCV2-RNA1-A 5′-ACAGAAGAGAGAA*A-3′; SCV2-RNA2-A 5′-GAGAGAGAGAACAGAAAAA*G-3′; RNA40 5′-GCCCGUCUGUUGUGUGACUC*U-3′ (*indicates a phosphorothioate linkage). In addition, in selected experiments, cells were stimulated with 0.2–10 µM R848 (InvivoGen, San Diego, CA, USA), 1 µg/mL ultrapure LPS (E. coli 0111:B4 strain, InvivoGen), or 20 ng/mL PMA (Sigma-Aldrich) and, where indicated, cells were pretreated for 1 h with 25 nM Bafilomycin A1 (Merck, Darmstadt, Germany) or 5–20 µM CU-CPT9a (Sigma-Aldrich).

### 2.2. Reverse-Transcription Quantitative Real-Time PCR (RT-qPCR)

After incubation, neutrophils were pelleted by centrifugation and then total RNA was extracted by RNeasy mini kit (QIAGEN, Venlo, The Netherlands) [19]. To completely remove any possible contaminating DNA, an on-column DNase digestion with the RNase-free DNase set (QIAGEN) was performed during total RNA isolation [19]. Purified RNA was reverse-transcribed into cDNA using PrimeScript™ RT Reagent kit (Takara Bio, Kusatsu, Japan), while qPCR was carried out using TB green^®^ premix Ex Taq ™ (Takara Bio). Sequences of gene-specific primer pairs (Thermo Fisher Scientific) are listed in Table 1. Data were calculated by Q-Gene software (https://www.gene-quantification.de/download.html, accessed on 28 September 2022) and expressed as mean normalized expression (MNE) units after *RPL32* or *GAPDH* normalization [23].

### 2.3. RNA Sequencing (RNA-Seq)

Libraries for transcriptome analysis were prepared using the Smart-seq2 protocol [24], as already described [19]. Briefly, 2 ng of total RNA were copied into first strand cDNA by reverse transcription and template-switching oligo (dT) primers and an LNA-containing template-switching oligo (TSO). The resulting cDNA was pre-amplified, purified, and tagmented with Tn5 transposase (kindly gifted by Dr. Luigi Scietti, European Institute of Oncology, Milan, 20139, Italy). cDNA fragments generated after tagmentation were gap-repaired, enriched by PCR, and purified to create the final cDNA library. Libraries were sequenced using 75 bp single-end sequencing parameters on the Illumina NextSeq 500 (Illumina, Cambridge, UK) at the Centro Piattaforme Tecnologiche (CPT) of the University of Verona.

### 2.4. Computational Analysis of RNA-Seq Data

Computational analysis of transcriptome datasets generated by Smart-seq2 has been performed using the bioinformatic pipeline utilized in a previous study [19], with minor modifications. Briefly, binary base call (BCL) files generated by the Illumina sequencer were converted into FASTQ files using bcl2fastq v2.20 software. After quality filtering, according to the Illumina pipeline, the contaminant adapters in the FastQ files were detected using FastQC v0.11.9. Then, adapters removal and base quality trimming were performed using Trim Galore! (http://www.bioinformatics.babraham.ac.uk/projects/trim_galore/, accessed on 28 September 2022) script with the length parameter set to 50. Trimmed reads were quantified using Kallisto quant [25] applying parameters -bias -single -l 200 -s 20. Transcript quantification obtained from Kallisto was combined to gene level using tximport packages v1.22.0. Gene counts were normalized among various samples using DESeq2 v1.34.0, and only genes coding to protein and long non-coding RNA (lnRNA) were retained for downstream analysis. DESeq2 was used to generate the expression metric and fragment per kilobase of transcript per million mapped reads (FPKM). To avoid possible noise of genes expressed at very low levels, only genes expressed above 1 FPKM in at least 1 sample were considered as “expressed” genes and retained for downstream analysis. Differentially expressed genes (DEGs) were identified using DESeq2, by using a selection parameter adjusted *p*-value lower than 0.01 and a Wald test or likelihood ratio test (LRT) for comparison, respectively, of two or more datasets. For k-means clustering analysis, the top 20% most variable genes in at least 1 of the conditions across stimulated and unstimulated neutrophils were considered. Gene expression FPKMs were log2-transformed, and for each gene the z score was calculated. Before clustering, the optimal number of clusters was estimated using the “clusGap” function of the R package cluster v2.1.3 [26]. Batch effects were removed using the limma package’s “removeBatchEffect” function before performing a principal component analysis (PCA). The PCA was performed on DEGs using the Bioconductor/R package pcaExplorer v.2.20.2. The DEGs of clusters identified by k-means clustering analysis were examined using the “EnrichedDAVID” function of ClusterProfiler [27], considering exclusively the enrichment of KEGG pathways. Single-sample gene set enrichment analysis (ssGSEA) scores were calculated using the bioconductor/R package “GSVA” (function gsva -arguments: method = “ssgsea”, mx.diff = TRUE) as previously described [28]. The ssgsea enrichment scores were generated for each gene set of the significantly enriched KEGG pathways using the vst-transformed counts by DESeq2 (function vst -arguments: blind = FALSE).

### 2.5. Production of Neutrophil Extracellular Trap (NET)

To visualize NET, isolated neutrophils (5 × 10^6^ cells/mL) were seeded on polylysine-coated glass slides and left adhering for 30 min at 37 °C. Then, neutrophils were stimulated directly on the glasses for 1 h. Next, cells were fixed with 1% paraformaldehyde (Thermo Fisher Scientific), and incubated with a rabbit anti-citrullinated–histone 4 antibody (Ab81797, Abcam, Cambridge, MA, USA), followed by an Alexa-488 conjugated anti-rabbit antibody (Thermo Fisher Scientific). Hoechst 33342 dye was used to counterstain cell DNA. NETs were visualized with the fluorescence microscopy Zeiss Observer.Z1 (Carl Zeiss, Oberkochen, Germany) at a magnification of 200× and Apotome2 for optical sectioning. Images were acquired using AxioVision Rel 4.8.Ink software (Carl Zeiss).

In addition, NET production was quantified by analyzing the DNA-associated elastase activity. For this purpose, neutrophils (2.5 × 10^6^ cells/mL) seeded in a 96-well culture plate were pretreated or not with CU-CPT9a for 30 min and then exposed to the stimuli indicated before. After 4 h, wells were washed to remove soluble elastase and DNAse I (1 U/mL) was added to digest extracellular DNA and free NET-adsorbed elastase. Finally, 10 min later, DNase activity was blocked by EDTA and supernatants were collected. Elastase activity in the supernatants was assessed using 0.5 mM of the fluorogenic elastase substrate (Z-Ala-Ala-Ala-Ala)2Rhodamine110 (Cayman Chemical, Ann Arbor, MI, USA) and fluorescence was monitored using a fluorescence plate reader.

### 2.6. Cytokine and Granule Protein Release

Analyte concentrations in cell-free supernatants were measured by commercially available ELISA kits, specific for human IL-1ra, elastase (R&D systems, Minneapolis, MN, USA), TNFα, IL-6 and CXCL8 (Mabtech, Nacka, Sweden). The lowest detection limits of these ELISA were: 39.1 pg/mL for IL-1ra, 46.9 pg/mL for elastase, 7.8 pg/mL for TNFα, 15.6 pg/mL for IL-6 and 7.8 pg/mL for CXCL8.

To measure lactoferrin we used a homemade developed immunoassay. Microtitration 96-well plates (Thermo Fisher Scientific) were coated overnight at 4 °C with 50 µL of supernatants from 5 × 10^6^/mL PMN culture, diluted 1:750 in 50 mM carbonate buffer, pH 9.6. The standard curve was prepared from recombinant lactoferrin (Sigma-Aldrich). The plate was then washed with PBS-0.1% Tween 20 (PBS-T) and blocked with 100 µL of PBS-Tween 0.1%, BSA 0.5% (blocking solution) for 1 h. After washing, biotinylated anti-human lactoferrin mAb (Sigma-Aldrich L-3262) was added in 1:2000 dilution in blocking solution and incubated for 1 h at RT. After washing, 50 µL of blocking solution containing streptavidin HRP anti-rabbit-PO (NA 934V, Cytiva) diluted 1:5000 was added, and the plates were incubated for 1 h at RT. Wells were further washed with PBS-T, and reactions were started by adding 50 µL of tetramethylbenzidine (TMB) solution (Sigma-Aldrich) and stopped by adding 50 µL of 1 M H_2_SO_4_. The absorbances (OD) of the samples were measured using a Victor3 multilabel reader (PerkinElmer, Shelton, CT, USA) at 450 nm and 550 nm.

### 2.7. Flow Cytometry

The expression of cell surface markers was evaluated by the use of fluorochrome-conjugated antibodies. 1 × 10^5^ neutrophils were centrifuged and suspended in 100 μL of PBS containing 2 mM EDTA and 2% of complemented-inactivated FCS (FACS Buffer). For FcγR blocking, 5% complemented-inactivated human serum was added to neutrophils suspension, for 5 min at RT. Neutrophils were then stained for 15 min at RT, or 30 min at 4 °C, with the following antibodies: FITC anti-human CD66b (clone GI0F5, BioLegend, San Diego, CA, USA), PE anti-human CD35 (REA1133, Miltenyi Biotec, Bergisch Gladbach, Germany), PE-vio770 anti-human CD11b (Miltenyi Biotec, REA713), APC anti-human CD62L (clone 145/5, Miltenyi Biotec), and APC-Cy7 anti-human CD16 (clone 3G8, BioLegend). Fluorochrome-conjugated antibodies were used at working dilutions as specified in the corresponding datasheets. Sample fluorescence was then measured by MACSQuant16 Analyzer (Miltenyi Biotec), while data analysis was performed using FlowJo software Version 10 from Tree Star (Ashland, OR, USA).

### 2.8. Neutrophil Viability 

Neutrophil viability was assessed by Vybrant/SYTOX staining. Briefly, after 6 h or an overnight treatment with the agonists indicated above, 1 × 10^5^ neutrophils were centrifuged at 300× *g* for 5 min, the medium was removed, and ultimately suspended in 100 μL Hank’s balanced salt solution (HBSS) buffer containing 5 µM Vybrant™DyeCycle™ Violet stain (Thermo Fisher Scientific) and 5 μM SYTOX™AADvanced™ dead cell stain kit (Thermo Fisher Scientific). Cells were then put on ice for 30 min, protected from light. Then cells were washed and suspended in 100 μL HBSS buffer. Finally, sample fluorescence was measured by MACSQuant16 Analyzer (Miltenyi Biotec). Cell viability was defined as the percentage of cells that were double negative for both stains (Vybrant/SYTOX, respectively).

### 2.9. Production of Superoxide Anion (O_2_^−^)

After isolation, neutrophils were suspended in HBSS buffer containing 80 μM ferricytochrome C type III (from bovine heart, C-7752, Sigma-Aldrich), 0.5 mM CaCl_2_, and 1 mg/mL glucose, and then distributed in a 96-well plate (1 × 10^5^ cells/100 μL/well) to be incubated for 10 min at 37 °C before stimulation. Plates were then incubated at 37 °C in an automated ELx808IU microplate reader (BioTek, Winooski, VT, USA) to record cytochrome C reduction, measuring at intervals of 5 min for 90 min the Δ O.D. 550 nm/465 nm. O_2_^−^ production was finally calculated using an extinction coefficient of 24.5 mM.

### 2.10. Statistical Analysis

Data are expressed as mean ± SEM of the number of indicated experiments. Where applicable, normality distribution was estimated using the D’Agostino–Pearson or Shapiro–Wilk normality test. Statistical evaluation for normally distributed data was performed by using one-way or two-way analysis of variance (ANOVA), followed by Tukey’s or Bonferroni’s post hoc test, respectively. Non-normally distributed data were assessed with Mann–Whitney test, or, for multiple group comparison, with Kruskal–Wallis or Friedman tests followed by Dunn’s multiple comparison test. The tests used are indicated in the respective figure legends. Values of *p* < 0.05 were considered statistically significant. Statistical analysis was performed by GraphPad Prism v.7.0 software.

## 3. Results

### 3.1. SCV2-RNAs Induce TNFα, CXCL8, and IL-1ra Production by Human Neutrophils

**Figure 1:**
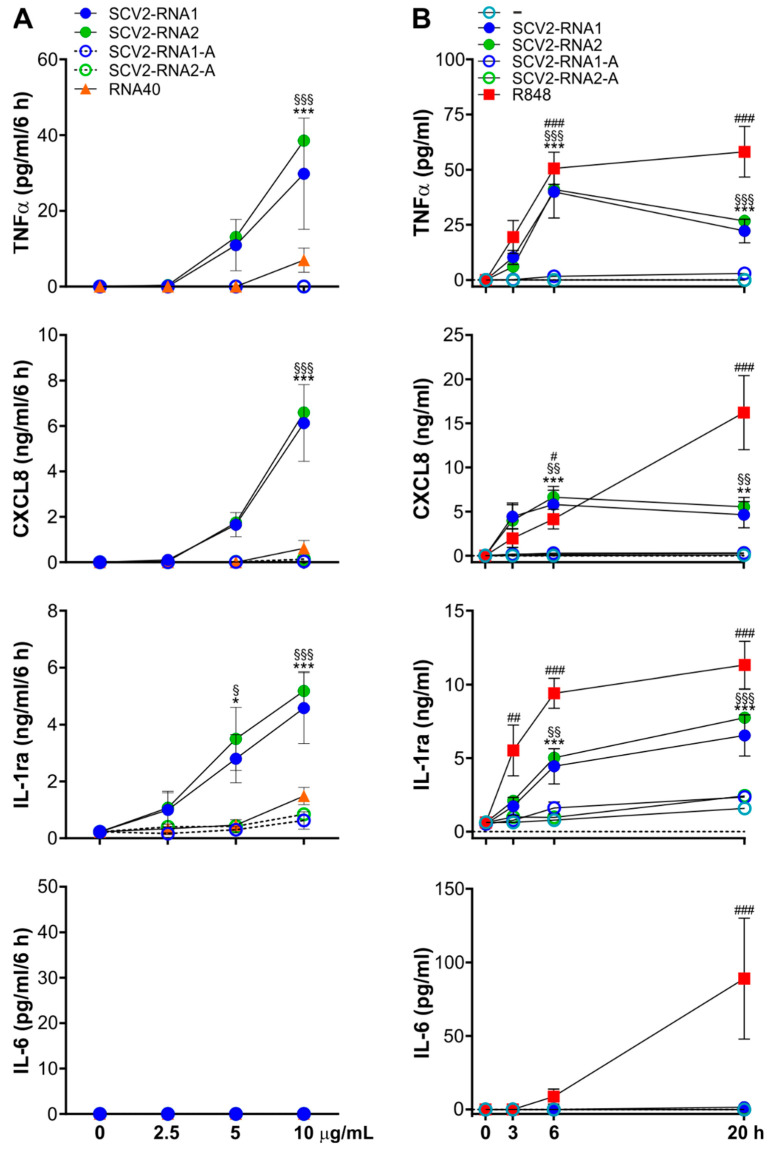
Cytokines production in neutrophils stimulated by SCV2-RNA1 and SCV2-RNA2. Production of TNFα, CXCL8, IL-1ra, and IL-6, by ssRNA-stimulated neutrophils in (**A**) dose-response and (**B**) time course experiments. In (**A**) neutrophils were stimulated for six hours with 2.5, 5 and 10 µg/mL of SCV2-RNA1, SCV2-RNA1-A, SCV2-RNA2, SCV2-RNA2-A, and RNA40. In (**B**) neutrophils were stimulated for three, six, and 20 h with 5 µM R848 and 10 µg/mL of SCV2-RNA1, SCV2-RNA1-A, SCV2-RNA2 and SCV2-RNA2-A. (**A**,**B**) Supernatants were collected, and cytokines levels were measured by ELISA. Results are expressed as the mean value ± SEM of n = 3–14 independent experiments. */§/# *p* < 0.05, **/§§/## *p* < 0.01, ***/§§§/### *p* < 0.001 by two-way ANOVA corrected for Tukey (**A**) or Dunnett’s (**B**) multiple comparisons test. § symbol indicates statistically significant differences between SCV2-RNA1 and SCV2-RNA1-A (**A**) or unstimulated cells (**B**), * symbol indicates statistically significant differences between SCV2-RNA2 and SCV2-RNA2-A (**A**) or unstimulated cells (**B**), # symbol indicates statistically significant differences between unstimulated cells and R848 (**B**).

Highly purified populations of human neutrophils (99.7 ± 0.2%) were incubated with increasing concentrations (up to 10 µg/mL) of either SCV2-RNA1 (5′-UGCUGUUGUGUGUUU-3′) or SCV2-RNA2 (5′-GUGUGUGUGUUCUGUUAUUG-3′) [21] and scrambled SCV2-RNA1 or SCV2-RNA2 sequences having uridines (U) replaced by adenines (A) (here named as SCV2-RNA1-A and SCV2-RNA2-A, respectively). As a control, neutrophils were also treated with RNA40, an RNA-oligonucleotide used as a prototypical sequence stimulating immune cells, corresponding to a GU-rich ssRNA sequence from the U5 region of HIV-1 genome [20]. We initially measured the extracellular concentrations of some of the cytokines known to be released by TLR8-activated neutrophils (i.e., CXCL8, TNFα, IL-1ra, and IL-6) [17,18,29]. As shown in Figure 1A, we found that the two ssRNAs were comparable in their effects, as 10 µg/mL of both SCV2-RNA1 and SCV2-RNA2 stimulated the release of TNFα, CXCL8, and IL-1ra at higher levels than 2.5 or 5 µg/mL. Extracellular IL-6 was not detected under all experimental conditions and as expected, SCV2-RNA1-A and SCV2-RNA2-A were ineffective in triggering cytokine production (Figure 1A). A total of 10 µg/mL SCV2-RNA1 and SCV2-RNA2 were then employed in all subsequent experiments since higher concentrations could not be used due to methodological issues. In fact, the transfection protocol establishes that if we increase the amount of SCV2-RNA, we must increase also the amount of DOTAP, thus exceeding its recommended concentration to avoid toxic effects.

By doing so, time-course experiments highlighted that, while TNFα and CXCL8 extracellular levels remained stable at time-points longer than six hours, IL-1ra release induced by SCV2-RNA1 and SCV2-RNA2-, but not SCV2-RNA1-A or SCV2-RNA2-A-treated neutrophils, slightly, but steadily, augmented (Figure 1B). However, confirming its potent effects on neutrophils [18], treatment of neutrophils with R848 resulted in a time-dependent production not only of TNFα, CXCL8, and IL-1ra (at higher levels than SCV2-RNA1 and SCV2-RNA2), but also of IL-6 (Figure 1B). In addition, RT-qPCR experiments confirmed that treatment of neutrophils with SCV2-RNA2 induces high levels of *CXCL8*, *TNF*, and *IL1RN* mRNAs, even though lower than those reached upon R848 stimulation (Supplementary Figure S1). RT-qPCR experiments also indicated that SCV2-RNA2 represents a poor inducer of *IL6* transcription (Supplementary Figure S1), therefore in accordance with the lack of IL-6 release in neutrophils incubated with SCV2-RNA2 (Figure 1B). Notably, no induction of mRNAs for type I interferons and interferon-stimulated genes (ISGs), such as *IFIT1* and *ISG15*, was observed in SCV2-RNA2-treated neutrophils (Supplementary Figure S1), similar to what previously reported for R848 [19]. Altogether, these data demonstrate that SCV2-RNAs promote the production of relevant amounts of TNFα, CXCL8, and IL-1ra by neutrophils, even with different kinetics and efficacy compared to those triggered by R848. 

### 3.2. SCV2-RNAs Delay Spontaneous Apoptosis of, as Well as Activate, Neutrophils

**Figure 2:**
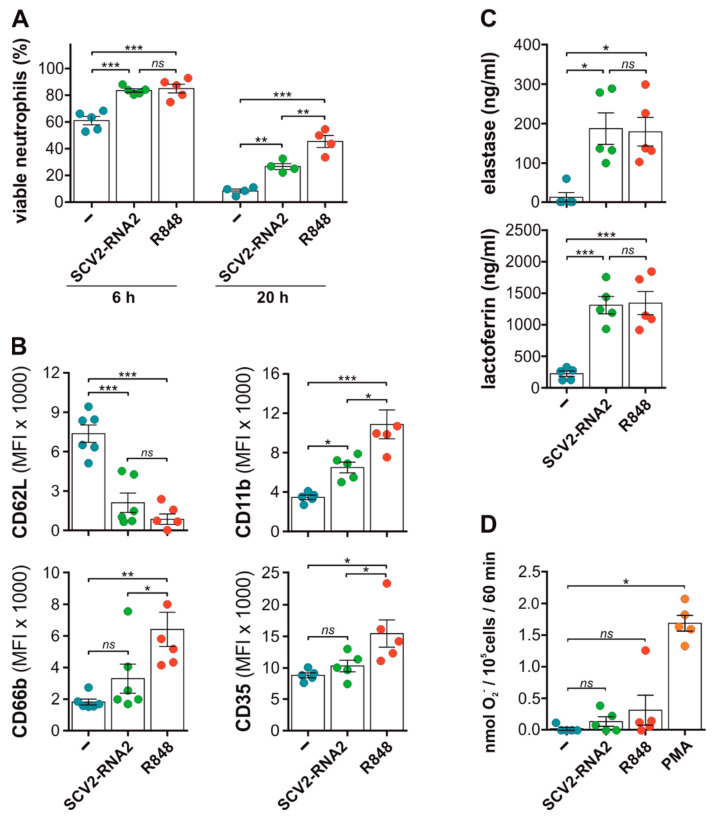
SCV2-RNA2 efficiently activates neutrophil effector functions. (**A**,**B**) Neutrophils were incubated for six (**A**,**B**) or 20 h (**A**) with or without 10 µg/mL SCV2-RNA2 or 5 µM R848. Cells were then collected, and neutrophil viability (**A**) and surface marker expression (**B**) were evaluated by flow cytometry. (**A**) Histograms show the percentage of viable cells (mean ± SEM, n = 4–5) defined as Vybrant™/Sytox™ double negative cell population (see Section 2). (**B**). After stimulation, neutrophils were incubated with specific fluorochrome-conjugated antibodies anti-CD66b, -CD11b, -CD62L, and -CD35 to evaluate their membrane expression by flow cytometry. Histograms show the median of the mean fluorescence intensity (MFI) ± SEM, obtained from n = 5–6 independent experiments. (**C**) Release of granule protein in stimulated neutrophils. Neutrophils were incubated for three hours with or without 5 µM R848 and 10 µg/mL SCV2-RNA2 and supernatants were collected. The release of elastase (upper panel) and lactoferrin (lower panel) was assessed by ELISA assays. Results are expressed as mean value ±SEM from n = 4–5 independent experiments. (**A**–**C**) * *p* < 0.05, ** *p* < 0.01, *** *p* < 0.001, analysis was performed using one-way ANOVA corrected for Holm–Sidak’s multiple comparison test. (**D**) Superoxide anion (O_2_^−^) production in SCV2-RNA2 stimulated neutrophils. Isolated neutrophils were left untreated or stimulated with 10 µg/mL SCV2-RNA2, 5 µM R848 and 20 ng/mL PMA. Histograms show the amount of nmol of O_2_^−^ produced by neutrophils after 60 min of stimulation, measured by cytochrome C reduction assay (see Section 2). Results are expressed as the mean value ±SEM of n = 5 independent experiments. ns (not significant) *p* > 0.05, * *p* < 0.05, ** *p* < 0.01, *** *p* < 0.001, by Kruskal–Wallis and Dunn’s post hoc test. (**A**–**D**) Results for every experiment are indicated by colored dots.

We then investigated the effects of SCV2-RNA1 or SCV2-RNA2 on apoptosis, expression of surface molecules (i.e., CD11b, CD62L, CD66b and CD35), production of superoxide anion, and release of granules by neutrophils. We found that, similar to but less efficiently than R848 [17,30,31], SCV2-RNAs reduce the degree of spontaneous apoptosis of neutrophils, an effect already significant after six hours of incubation (Figure 2A and Supplementary Figure S2A). These data are consistent with a more transient stimulatory capacity of neutrophils by SCV2-RNAs than R848, as observed in the case of their TNFα and CXCL8 production (Figure 1B). Concerning surface molecules, we observed a strong decrease in CD62L expression in neutrophils treated with SCV2-RNAs, at levels comparable with those found after stimulation with R848 (Figure 2B and Supplementary Figure S2B). By contrast, SCV2-RNAs did not significantly change the expression of CD35 or CD66b, as opposed to the effects of R848 (Figure 2B and Supplementary Figure S2B). CD11b was instead increased in SCV2-RNAs-treated neutrophils compared with untreated ones (Figure 2B and Supplementary Figure S2B), although at levels lower than those observed in R848-stimulated cells, with SCV2-RNAs-A being ineffective in modulating marker expression (Supplementary Figure S2B). Since SCV2-RNA1 and SCV2-RNA2 give similar results in all assays performed (Figure 1 and Supplementary Figure S2), we decided to use only SCV2-RNA2 in subsequent experiments. SCV2-RNA2 was found to promote a rapid extracellular release of elastase and lactoferrin (markers of azurophilic and specific granules, respectively) by neutrophils, at similar levels to those induced by R848 (Figure 2C). Finally, both SCV2-RNA2 and R848 were found to be very poor stimuli for superoxide anion release, unlike phorbol 12-myristate 13-acetate (PMA), used as control agonist (Figure 2D). Altogether, these data show that SCV2-RNA2, in addition to TNFα, CXCL8, and IL-1ra production, triggers several neutrophil effector functions.

### 3.3. SCV2-RNA2 and R848 Similarly Modify the Transcriptomic Profile in Human Neutrophils

**Figure 3:**
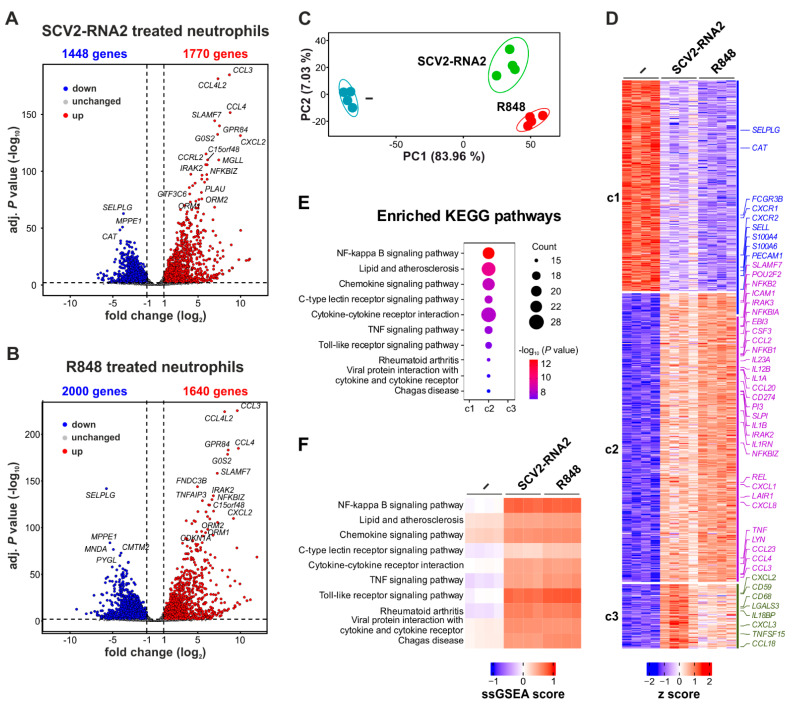
Gene expression profile of neutrophils treated with SCV2-RNA2 or R848. (**A**,**B**) Volcano plot displaying differentially expressed genes (DEGs) in neutrophils incubated with SCV2-RNA2 (**A**) or R848 (**B**) for six hours. Each dot in the plot represents a single DEG. DEGs showing significantly increased or decreased expression (*p* < 0.01, calculated by Wald’s test) are marked by red and blue dots, respectively, while genes not significantly modulated by stimulation are shown as grey dots. (**C**) A PCA scatterplot based on the DEGs identified among neutrophils incubated with or without SCV2-RNA2 or R848 for six hours. Blue, red, and green circles represent, respectively, samples from resting, R848-stimulated, and SCV2-RNA2-stimulated cells (n = 4). (**D**) Heatmap displaying the expression patterns of the gene clusters (c1–c3) resulting from the k-means clustering analysis of DEGs. Relative expression levels for a single transcript were calculated by z score. Selected genes of each cluster are depicted on the right y axis. (**E**) KEGG pathways enriched by genes associated with the gene cluster c2. No statistically significant enriched KEGG pathways were present in c1 and c3. The top 10 KEGG pathways with Benjamini–Hochberg-corrected *p* values < 0.05 (one-sided Fisher’s exact test) are shown. ‘Counts’ indicate the fraction of DEGs present in the given KEGG pathway. (**F**) Heatmap representations of gene set variation analysis (GSVA) comparisons among untreated and SCV2-RNA2- or R848-treated neutrophil. Gene set signatures were obtained from KEGG pathways enriched in c2 (**E**). Color intensity of the squares is indicative of the GSVA score, which varies from 1 (maximal signature enrichment, indicated by red) to −1 (absent signature enrichment, indicated by blue).

We have previously shown that R848 represents a very powerful agonist for neutrophils, able to trigger remarkable chromatin remodeling and transcriptomic changes [19]. We thus performed RNA-seq experiments of neutrophils incubated with either SCV2-RNA2 or R848 for six hours, to compare their effects at the mRNA expression level. By pairwise comparison of gene expression data, we identified an almost equivalent number of upregulated genes (more than 1600, by a two-fold difference and adjusted *p* < 0.01) in SCV2-RNA2- and R848-treated neutrophils (Figure 3A,B). Accordingly, neutrophils were found to substantially accumulate the same over-expressed transcripts (i.e., with the highest fold-change) in response to the two stimuli, for instance, those encoding for chemokines (such as *CCL3, CCL4, CCL4L2,* and *CXCL2*), orosomucoid (such as *ORM1* and *ORM2*), and pro-inflammatory (such as *NFKBIZ* and *IRAK2*) proteins, as well as immunosuppressive molecules (such as *SLAMF7*) (Figure 3A,B). By contrast, the number of downregulated genes differed between the two stimuli (2000 vs 1448 in response to R848 and SCV2-RNA2, respectively) (Figure 3A,B). Next, we applied the likelihood ratio tests (LRTs) to the RNA-seq data and performed a principal component analysis (PCA) to better characterize the degree of similarity of the transcriptional programs activated by SCV2-RNA2 and R848. As shown in Figure 3C, while PC1 confirms net transcriptome differences between unstimulated and either SCV2-RNA2- or R848-treated neutrophils, PC2 (which was found to account for only seven percent variability) highlighted only minimal differences between the two stimuli. To further characterize these minimal differences, we performed a k-means clustering analysis and, in turn, identified three main clusters of DEGs (c1–c3), with c1 and c2/c3 including, respectively, downregulated and upregulated genes after stimulation. In the latter case, c2 and c3 consisted of genes more strongly upregulated by, respectively, R848 than SCV2-RNA2, and SCV2-RNA2 than R848 (Figure 3D). Among the DEGs present in the different clusters, we found that c1 was enriched with genes encoding for membrane proteins involved in the recruitment of neutrophils at the inflammatory site (such as *CXCR1*, *CXCR2*, *PECAM1*, *SELL*, and *SELPLG*), while c2 was found enriched with pro-inflammatory cytokine mRNAs (such as *TNF*, *IL1B*, *IL12B*, *EBI3*, *CXCL8*, *CCL3*, *CCL4*, *CCL23,* and others) and genes involved in NF-κB signalling (such as *NFKB1*, *NFKB2*, *REL*, *NFKBIA*, *NFKBIZ*, and *TNFAIP3*). In accordance, the “NF-kappa B signaling pathway” was the most enriched KEGG pathway of c2 (Figure 3E). As expected, gene set variation analysis (GSVA) revealed that the mRNA expression of the genes included in the enriched KEGG pathways of c2 was induced by both SCV2-RNA2 and R848 (Figure 3F). These results were also confirmed by gene ontology (GO) analysis, which pointed to “inflammatory response” and “cytokine production” as the most enriched GO terms in c2. We also found genes involved in the resolution of inflammation in c2, such as those for protease inhibitors (*PI3* and *SLPI*), immunosuppressive molecules (*SLAMF7*, *LAIR1* and *CD274*), as well as *POU2F2*, also known as OCT2, a transcription factor that we recently found to be involved in the amplification of the transcriptional response to TLR8-mediated activation [19]. In accordance, several OCT2-regulated genes, such as *IRAK3*, *NFKBIZ*, *LYN*, and *CXCL8* [19], were found present in c2. c3 genes were found to be more heterogeneous, but some of them were clearly involved in the inflammatory process, including cytokines and chemokines (such as *TNFSF15*, *CXCL2*, *CXCL3*, and *CCL18*), a cytokine receptor (*IL1R1*), a scavenger receptor (*CD68*), an inhibitor of the complement membrane attack complex (*CD59*), and an inhibitor of IL-18 signaling (*IL18BP*) (Figure 3D). Notably, no induction of ISGs was observed in SCV2-RNA2-treated neutrophils, confirming RT-qPCR results (Supplementary Figure S1) [19]. Altogether, these data indicate that, in neutrophils, SCV2-RNA2 activates a transcriptional program that almost overlaps with that observed in R848-treated cells.

### 3.4. The Transcriptomic Profile Induced by SCV2-RNA2 in Neutrophils Is Dependent on TLR8 Activation

**Figure 4:**
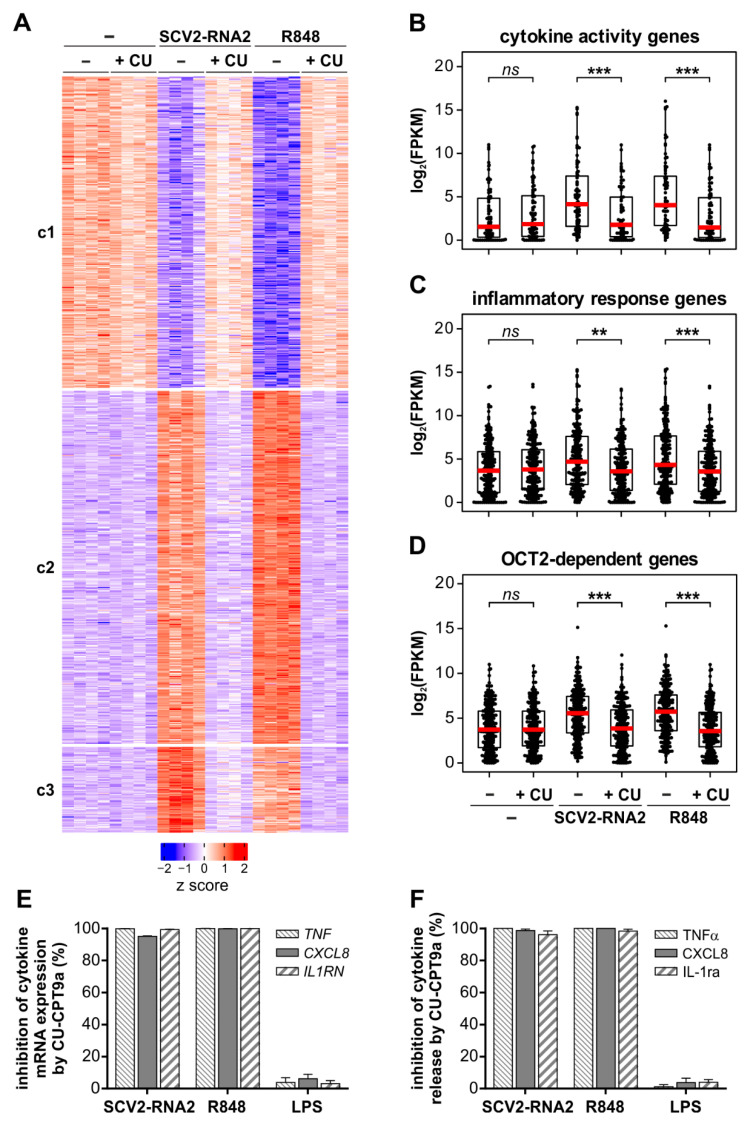
Effects of TLR8 inhibition in the transcriptome profiles of SCV2-RNA2 or R848 treated neutrophils, in cytokine mRNA expression and release. Neutrophils were stimulated with 10 µg/mL SCV2-RNA2 and 5 µM R848 in the presence or absence of the TLR8-specific inhibitor CU-CTP9a (10 µM) and subjected to RNA-seq analysis. (**A**) Heatmap displaying the expression patterns of the gene cluster (c1–c3) resulting from the k-means clustering analysis of DEGs from Figure 3D. Relative expression levels for a single transcript were calculated by z score. (**B**–**D**) Box plots showing the distribution of mRNA expression levels [as log2(FPKM + 1)] for genes associated with the GO terms “cytokine activity” (**B**) and “inflammatory response” (**C**), and for OCT2-dependent genes (**D**). The box plot shows the median (red line) with the lower and upper quartiles representing a 25th to 75th percentile range. (**B**–**D**) Asterisks stand for significant inhibition caused by CU-CPT9a treatment (ns *p* > 0.05, ** *p* < 0.01,*** *p* < 0.001 by Wilcoxon signed-rank test). (**E**,**F**) Neutrophils were pretreated for 30 min with 20 μM CU-CPT9a and then incubated for six hours with 5 μM R848, 10 µg/mL of SCV2-RNA2, or 1 μg/mL LPS. Cells were then lysed for RNA extraction (**E**) and cell-free supernatant was collected for evaluation of cytokine release (**F**). The mRNA expression and release (**F**) of TNFα, CXCL8, and IL-1ra were measured by RT-qPCR and ELISA, respectively. Graphs depict the percentage of inhibition exerted by CU-CPT9a expressed as mean ± SEM, n = 4.

We then performed new RNA-seq experiments, in which neutrophils were pre-treated with 20 µM CU-CPT9a, a specific TLR8 inhibitor [32], and then incubated with SCV2-RNA2 and R848 for six hours. These experiments had the purpose to clarify whether the effects triggered in neutrophils by SCV2-RNA2 (and R848 as well) depend on TLR8 activation. As shown in Figure 4A, this was found to be the case, as the modulatory effects on neutrophil gene expression determined by SCV2-RNA2 and R848 were found fully abolished by CU-CPT9a. Accordingly, increased expression levels of the genes belonging to the “cytokine activity” (Figure 4B) and “inflammatory response” (Figure 4C) GO terms (potentially related to severe COVID-19 pathogenesis) were completely suppressed by CU-CPT9a pre-treatment. Similarly, upregulation of OCT2-dependent genes was completely blocked by TLR8 inhibition (Figure 4D), while CU-CPT9a was found not to exert any relevant effects on the transcriptome of resting neutrophils (Figure 4A). Moreover, mRNA expression (Figure 4E) and release (Figure 4F) of TNFα, CXCL8 and IL-1ra by neutrophils treated for six hours with either SCV2-RNA2 or R848, but not LPS as expected, were completely abrogated by CU-CPT9a pre-treatment. Similar results were also obtained by pre-treatment of neutrophils with either M5049 (a potent small-molecule inhibitor of TLR7/8) [33] or Bafilomycin A1 (a drug known to block endosome acidification) [34]. All in all, data demonstrate that the whole transcriptomic reprogramming of human neutrophils determined by SCV2-RNA2 requires TLR8, as validated by the results on cytokine release.

### 3.5. SCV2-RNA2 Potently Induces the Release of NETs by Human Neutrophils in a TLR8-Dependent Manner

**Figure 5:**
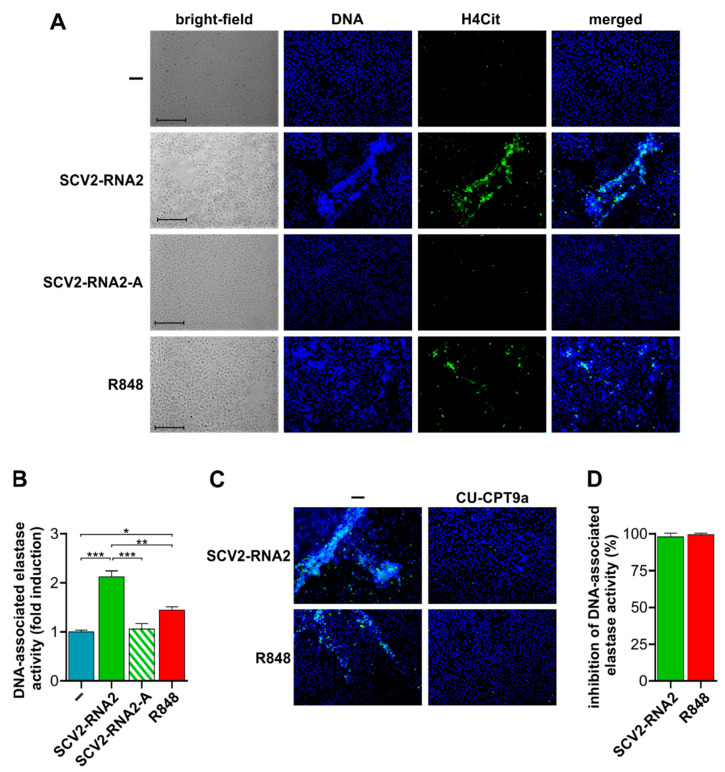
SCV2-RNA2 efficiently stimulates neutrophil extracellular trap (NET) formation which is blocked by TLR8 inhibition. Isolated neutrophils were left untreated (-) or stimulated with 10 µg/mL SCV2-RNA2, 10 µg/mL SCV2-RNA2-A, or 5 µM R848 for one hour (**A**) and four hours (**B**). (**A**) NETs were identified, using fluorescence microscopy, by the co-localization of citrullinated histone-4 (H4Cit) stained in green (AF-488) and DNA stained in blue (Hoechst). Bright-field images are also shown. Scale bar: 100 µm. (**B**) NETs were also quantified in cell supernatants by analyzing DNA-associated elastase activity after a limited DNase I digestion. Elastase activity associated with DNA was quantified by a fluorogenic elastase substrate and monitored with a fluorescent plate reader. Results are expressed as fold induction of elastase activity compared to unstimulated cells. * *p* < 0.05, ** *p* < 0.01, *** *p* < 0.001, one-way ANOVA followed by Tukey’s post hoc test. (**C**,**D**) Isolated neutrophils were pre-treated with or without 5 μM CU-CTP9a for 30 min and left untreated or stimulated with 10 µg/mL SCV2-RNA2 or 5 µM R848 for one hour (**C**) or four hours (**D**). (**C**) Merged images of NETs as identified in panel **A**. Scale bar: 100 µm. Representative experiments out of three. (**D**) NETs were quantified in cell supernatants by analyzing DNA-associated elastase activity after a limited DNase I digestion. Graphs depict the percentage of inhibition exerted by CU-CPT9a expressed as mean ± SEM, n = 3.

Finally, we assessed whether SCV2-RNA2 also triggers the release of NETs, one of the hallmarks of COVID-19 [35]. For these experiments, neutrophils were cultured in the absence of FCS since, under in vitro experimental conditions, FCS prevents NET formation [36]. As shown by fluorescence microscopy experiments (Figure 5A), neutrophils incubated with SCV2-RNA2, but not SCV2-RNA2-A, emit characteristic NETs structures, with long branches of extracellular DNA (seen by Hoechst staining) co-localizing with citrullinated histone 4 (H4Cit). Surprisingly, such a release of NETs resulted higher in response to SCV2-RNA2 than to R848 (Figure 5A). In fact, by precisely quantifying the release of NETs, we found that R848 had a poor effect, while SCV2-RNA2, but not SCV2-RNA2-A, doubled the amounts of DNA-associated elastase compared with unstimulated cells (Figure 5B). 

We then evaluated whether CU-CPT9a could also block NET release by neutrophils incubated with SCV2-RNA2. As demonstrated by fluorescence microscopy experiments, we found that TLR8 inhibition unequivocally prevents NET formation in response to either SCV2-RNA2 or R848 (Figure 5C). In fact, similar to unstimulated cells, no H4Cit positivity could be detected in neutrophils pre-treated with CU-CPT9a and then stimulated with SCV2-RNA2. Data were confirmed also by measuring the enzymatic activity of DNA-associated elastase in cell culture supernatants (Figure 5D), as CU-CPT9a treatment was found to inhibit NET release not only by SCV2-RNA2- but also by R848-treated neutrophils (Figure 5C,D). Altogether, these findings clearly demonstrate that SCV2-RNA2 can act as a potent inducer of NET release, and that, again, they require the involvement of TLR8.

## 4. Discussion

The SARS-CoV-2 genome contains several hundred GU-rich sequences that might function as agonists for TLR7 and TLR8, which are known to bind ssRNA. Two of these representative sequences (that we named SCV2-RNA1 and SCV2-RNA2) were synthesized in vitro and shown by us to potently stimulate human DCs in terms of IFN and proinflammatory cytokine production, as well as Th1 polarization [21]. Moreover, in vivo experiments demonstrated that SCV2-RNA1 and SCV2-RNA2 induce MyD88-dependent lung inflammation and phenotypical maturation of splenic DCs in mice [21]. In this study, we report that SCV2-RNA2 represents a potent stimulus for human neutrophils, in which it triggers cytokine mRNA expression and production, surface molecule modulation, degranulation and, under appropriate experimental conditions, formation of NETs. Moreover, by using a specific TLR8 inhibitor, named CU-CPT9a [32], we prove that all SCV2-RNA2-induced functional activities of neutrophils are strictly mediated by TLR8-dependent signaling. Therefore our data arein favor of the use of TLR8 inhibitors as integrative therapy in fighting the exaggerated inflammatory response observed in severe COVID-19 patients [37]. In this context, a clinical trial for the treatment of COVID-19 pneumonia patients is currently ongoing (https://www.merckgroup.com/en/news/m5049-treatment-covid-19-pneumonia.html, accessed on 28 September 2022), and takes advantage of M5049 (from MERCK) which displays dual TLR7/8 antagonist activity. 

Another remarkable observation of our study is the demonstration that SCV2-RNA2 promotes transcriptomic modifications in neutrophils that, within six hours of incubation, mostly overlap those observed to occur in response to R848. The latter is a TLR7/TLR8 agonist that is known to potently activate neutrophils [18,19]. Transcriptomic changes in response to SCV2-RNA2 include, for instance, an upregulation of mRNA expression for several cytokines and chemokines, such as TNFα, CXCL8, IL-1ra, IL-1β, G-CSF, IL23, EBI3, and CCL23, which we previously reported to be secreted by R848-stimulated neutrophils [38]. In this study, in fact, we selectively focused on TNFα, CXCL8, and IL-1ra production by SCV2-RNA2-treated neutrophils. It is likely that cytokines expressed and produced by SCV2-RNA2-activated neutrophils, and/or other cell types, such as DCs [21], contribute to the “cytokine storm” observed in severe COVID-19 disease [10]. Confirming results obtained with R848, no evidence for the production of type I IFNs [18,19], or for type II IFNs and IL-12 [30], were observed in SCV2-RNA2-treated neutrophils. Alongside cytokines and chemokines, SCV2-RNA2 was found to induce the transcription of thousands of pro-inflammatory genes, among which we found *POU2F2/OCT2*, a transcription factor that we have recently proved to act as a transcriptional amplifier in R848-activated neutrophils [19]. In accordance, we observed an increased expression of OCT2-dependent genes in SCV2-RNA2-treated neutrophils, including *IRAK3*, *NFKBIZ*, *LYN*, and *CXCL8*. Moreover, by k-means clustering analysis, we noticed a cluster of genes induced at higher levels by SCV2-RNA2 than R848, and vice versa a cluster of genes induced at higher levels by R848 than SCV2-RNA2. More relevant differences between SCV2-RNA2 and R848 were observed at the 20 h time point of neutrophil treatment, at which only R848 was confirmed to stimulate the release of IL-6 [18,19], as well as much higher amounts of CXCL8 and IL-1ra than SCV2-RNA2. Since human neutrophils require a strong and prolonged stimulation to become able to produce IL-6, as they need to de novo express the IκBζ coactivator and rearrange the chromatin [18,19], it is plausible that, at least in neutrophils, SCV2-RNA2 is unable to sustain TLR8-activation because it is likely degraded by the high RNAase content. Consistently, we observed that only R848, not SCV2-RNA2, promotes an efficient survival of neutrophils after 20 h of incubation, while both SCV2-RNA2 and R848 were found to exert a similar prolonged neutrophil viability after six hours of cell incubation. Another explanation for the different effects that these two agonists have on TLR8 is suggested by crystallographic studies of TLR8 complexes bound to imidazoquinoline or ssRNA [39,40]. In fact, R848, an imidazoquinoline derivative, is a highly stable small synthetic compound, while SCV2-RNAs are ssRNA+ with phosphorothioate linkages that simply protect it from degradation. To activate TLR8, ssRNA is degraded and recognized by two different binding sites in the receptor: the first site recognizes U mononucleoside, while the second site binds oligonucleotides which greatly increase the affinity of U for TLR8. Conversely, small antiviral compounds, such as R848, bind only to the first site and exhibit sufficiently high affinity to activate TLR8 by themselves [39,40]. Hence, even though we cannot exclude that stimulation of neutrophils with higher concentrations of SCV2-RNA2 would have promoted the same stimulatory effects of R848, we would also speculate that increased degradation of SCV2-RNA2 at a delayed incubation time likely reduces the availability of ssRNA and consequently has a lower affinity of TLR8 for U. Whatever the case, no induction of ISGs was observed in neutrophils treated with SCV2-RNAs, suggesting no production of type I interferon, similar to R848-stimulated neutrophils [18,19]. By contrast, type I interferons were observed to be released extensively by pDCs incubated with SCV2-RNAs. However, SCV2-RNAs specifically trigger TLR7 in this cell type and therefore are not blocked by CU-CTP9a [21]. Interestingly, in COVID-19 patients the high levels of plasma type I interferon and the low levels of pro-inflammatory cytokines are associated with mild symptoms and a good prognosis [41]. In patients with severe disease, instead, while type I interferon levels remain high, the amounts of pro-inflammatory cytokines are substantially increased [41]. We suppose that the use of a TLR8-specific inhibitor (i.e., CU-CTP9a), rather than a pan TLR7/8 inhibitor (i.e., M5049), would be theoretically more beneficial for patients, since a TLR8-specific inhibitor would selectively block the exaggerated production of pro-inflammatory cytokines and NET formation exclusively by neutrophils, myeloid DC and monocytes, but not the advantageous production of type I IFNs by pDCs. 

Our data raise the question on how/when SCV2-RNAs would activate neutrophils in vivo. Viral recognition by endosomal TLRs can take place independently of infection because of pathogen endocytosis [42]. In addition, whether SARS-CoV-2 directly infects neutrophils or is instead phagocytosed is still a matter of discussion. SARS-CoV-2 is known to infect target cells via two surface receptors, namely ACE2 and CD147 [43]. The latter is expressed on the surface of neutrophils from healthy donors and is upregulated in COVID-19 patients [44], but no studies have shown that CD147 can specifically mediate neutrophil infection by SARS-CoV-2 yet. Contrasting data have been instead reported concerning ACE2 expression by neutrophils, with some authors [45,46], but not others [47,48], detecting it. Recently, it has been reported that SARS-CoV-2 enters monocytes via CD16, whereas no detection of the virus was observed in neutrophils incubated with SARS-CoV-2 [49], except in one study [47]. Studies performed by scRNA-seq of immune cells isolated from bronchoalveolar lavage fluid (BALF) of COVID-19 patients demonstrated the presence of SARS-CoV-2 RNA in neutrophils [46,50]. These conflicting results illustrate the need for definitive studies to clarify whether SARS-CoV-2 directly infects neutrophils (and more broadly innate immune cells) and/or whether it is instead phagocytosed by the same cells.

In any case, high concentrations of NET markers, such as cell-free DNA, citrullinated histones and granule proteins associated with DNA, have been detected in the plasma of severe COVID-19 patients [51]. In addition, neutrophils isolated from COVID-19 patients were found to display a spontaneous release of NETs ex vivo [35]. In this context, C5a, viral-antigens/antibodies complexes, and endogenous IL-6, CXCL8, or G-CSF have been proposed as potential mediators of NET formation in vivo [52]. It is conceivable that an imbalance between the release of NETs and NETs degradation might lead to a NET-induced platelet aggregation and microthrombi formation in COVID-19 patients [53]. Interestingly, in vitro treatment of healthy neutrophils with viable SARS-CoV-2 virus was shown to directly promote the release of NETs [47,54], similar to what was observed by neutrophil incubation with other RNA viruses, such as HIV [55] and chikungunya virus [56]. It should be pointed out here that Veras et al. [47] suggested that NET formation is mediated by a TLR7-dependent recognition of SARS-CoV-2 RNA. However, human neutrophils do not express TLR7 [13,18,57], which explains why we show that SCV2-RNA2 induces the release of NETs by TLR8-dependent recognition. 

The findings that SCV2-RNAs promote either survival of neutrophils or their NET formation, in principle two opposite effects, are actually explained by the different experimental conditions utilized to investigate these responses in vitro. In fact, apoptosis studies necessitate the use of medium containing FCS [58], while NETosis experiments require serum-free medium [36]. Whether in vivo SCV2-RNAs trigger one or the other is likely determined by the microenvironment in which neutrophils detect ssRNA (blood, infected tissue, or other sites). It is also possible that the induction of NETosis by SCV2-RNAs is not in contrast with the maintenance of neutrophil viability. In fact, as NETosis is a process distinct from either necrosis or apoptosis, at least two types of NETosis have been described for neutrophils, namely classical or suicide lytic NETosis and live cells or vital NETosis. Classical NETosis is ROS dependent while vital NETosis is not and it does not impact on neutrophil lifespan. The latter is rapid and it has been reported to be activated by different TLR ligands [59]. Considering that, in this study, NETosis observed in SCV2-RNA2-treated neutrophils is detected after one hour and that, neither SCV2-RNA2 nor R848 induce ROS production, we could hypothesize that TLR8 ligands may induce a vital NETosis. Obviously, further studies are required to ascertain this kind of occurrence.

In sum, by extending to neutrophils the results previously obtained in DCs [21], herein we confirm that SCV2-RNAs may act as potent agonists for innate immune cells. Our data also highlight that the recognition of GU-rich sequences of the SARS-CoV-2 genome function as specific TLR8 ligands in neutrophils, in turn indicating that the use of specific TLR8 inhibitors might serve for counteracting the excessive inflammatory responses observed in severe COVID-19 patients, at least those promoted by neutrophils.

## Figures and Tables

**Table 1:** Sequences of human gene-specific primer pairs used in RT-qPCR.

Gene	Forward Primers	Reverse Primers
*GAPDH*	AACAGCCTCAAGATCATCAGC	GGATGATGTTCTGGAGAGCC
*RPL32*	AGGGTTCGTAGAAGATTCAAGG	GGAAACATTGTGAGCGATCTC
*IL1RN*	TTCCTGTTCCATTCAGAGACGAT	AATTGACATTTGGTCCTTGCAA
*IL6*	GGCACTGGCAGAAAACAACC	GCAAGTCTCCTCATTGAATCC
*TNF*	GAGCACTGAAAGCATGATCC	CGAGAAGATGATCTGACTGCC
*CXCL8*	CTGGCCGTGGCTCTCTTG	CCTTGGCAAAACTGCACCTT
*IFNA(all)*	GTGAGGAAATACTTCCAAAGAATCAC	TCTCATGATTTCTGCTCTGACAA
*IFNB1*	CAGCAATTTTCAGTGTCAGAAGC	TCATCCTGTCCTTGAGGCAGT
*IFIT1*	TCATCAGGTCAAGGATAGTCTG	GGTGTTTCACATAGGCTAGTAG
*ISG15*	ACTCATCTTTGCCAGTACAGGAG	CAGCATCTTCACCGTCAGGTC

## Data Availability

Raw datasets have been submitted to the Gene Expression Omnibus database (http://www.ncbi.nlm.nih.gov/geo/, accessed on 28 September 2022) and are available under the accession number GSE214247.

## Supplementary Materials

The following supporting information can be downloaded at: https://www.mdpi.com/article/10.3390/cells11233785/s1, Figure S1: Cytokine gene expression in neutrophils stimulated with SCV2-RNA2; Figure S2: Flow cytometry analysis of SCV2-RNA1 and SCV2-RNA2 stimulated neutrophils to evaluate their viability and surface markers expression.
