# Supplementary material for: SARS-CoV-2-Associated ssRNAs Activate Human Neutrophils in a TLR8-Dependent Fashion
Source: Cells. 2022 Nov 26;11(23):3785. doi: 10.3390/cells11233785 (PMC9738506; doi:10.3390/cells11233785)
Supplement: Supplementary file 1 [file cells-11-03785-s001.zip › cells-1967303-supplementary.pdf]

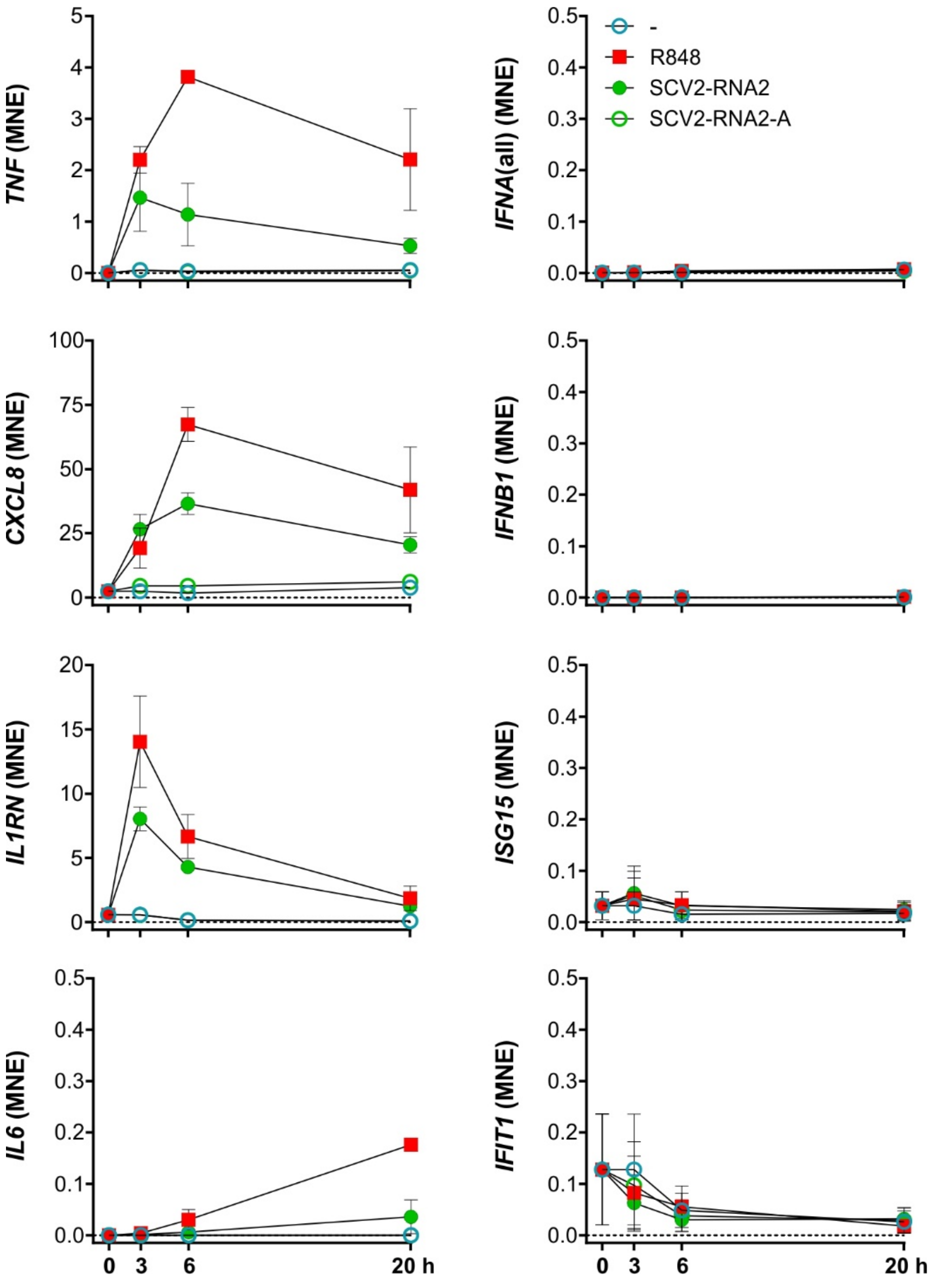

Supplementary Figure S1: Cytokine gene expression in neutrophils stimulated with SCV2-RNA2. (A) 5 × 10^6/ml highly pure neutrophils were incubated for up to 20 h in the presence or the absence of 10 µg/mL SCV2-RNA2, 10 µg/mL SCV2-RNA2-A or 5 µM R848. Plots show kinetics of *TNF*, *CXCL8*, *IL1RN*, *IL6*, *IFNA*(all), *IFNB*, *ISG15* and *IFIT1* mRNA expression, as measured by RT-qPCR. Results from a representative experiment out of three are depicted as mean normalized expression (MNE) units after normalization to *GAPDH* mRNA. Error bars represent SEs calculated from triplicate qPCR reactions.

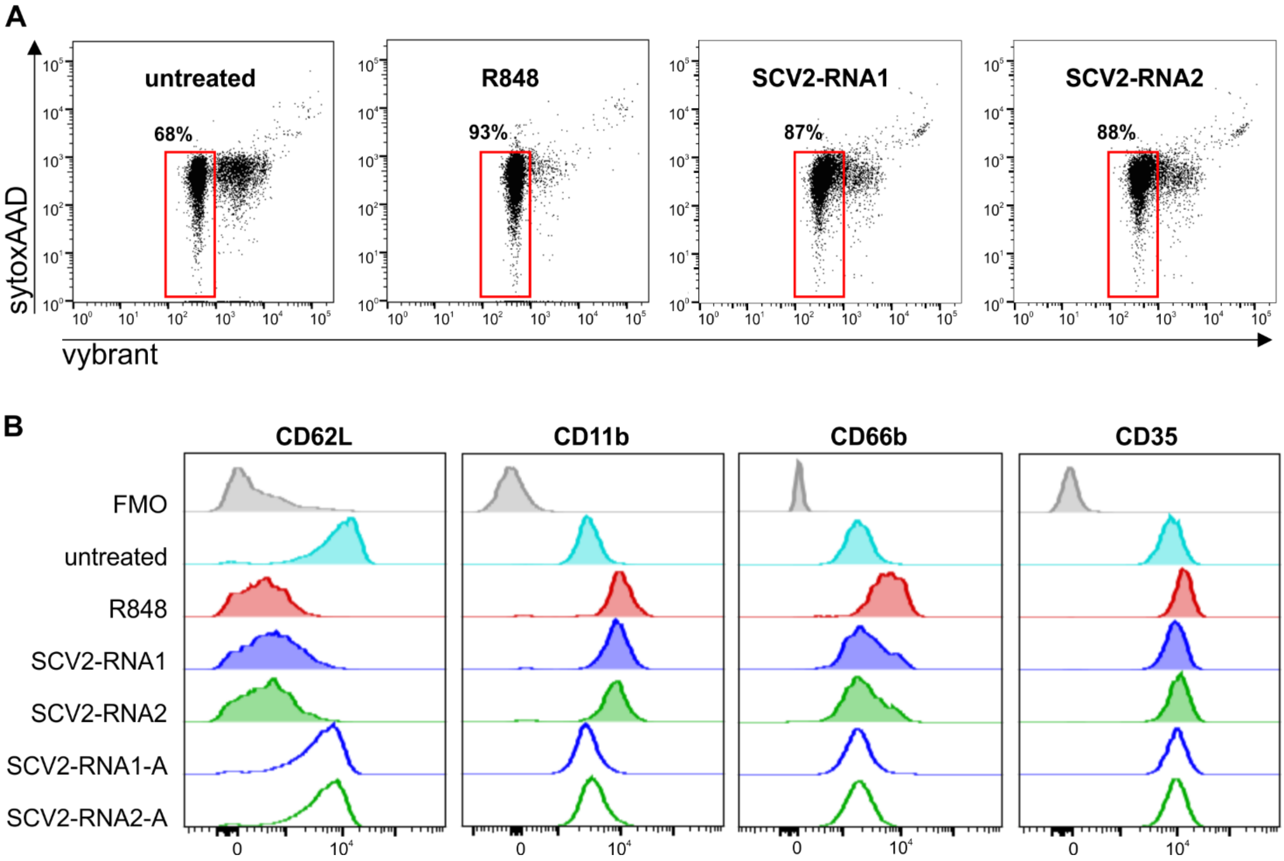

Supplementary Figure S2: Flow cytometry analysis of SCV2-RNA1 and SCV2-RNA2 stimulated neutrophils to evaluate their viability and surface markers expression. (**A**,**B**) Neutrophils were incubated for six hours with or without and 10 µg/mL SCV2-RNA1, 10 µg/mL SCV2-RNA2, 10 µg/mL SCV2-RNA1-A, 10 µg/mL SCV2-RNA2-A or 5 µM R848. Cells were then collected, and neutrophils viability (**A**) and surface markers expression (**B**) was evaluated by flow cytometry. (**A**) Dot plots showing the percentage of viable cells (red box) defined as Vybrant™/Sytox™ double negative cell population (see Section 2). (**B**) Histograms showing staining by specific anti-CD66b, -CD11b, -CD62L and -CD35 abs to evaluate their membrane expression. Representative experiments out of 5 and 3 are shown in panels (**A**) and (**B**), respectively
